# Non-invasive CT-derived fractional flow reserve and static rest and stress CT myocardial perfusion imaging for detection of haemodynamically significant coronary stenosis

**DOI:** 10.1007/s10554-019-01658-x

**Published:** 2019-07-04

**Authors:** Brian S. Ko, Jesper J. Linde, Abdul-Rahman Ihdayhid, Bjarne L. Norgaard, Klaus F. Kofoed, Mathias Sørgaard, Daniel Adams, Marcus Crossett, James D. Cameron, Sujith K. Seneviratne

**Affiliations:** 1 Monash Cardiovascular Research Centre, Monash University and MonashHeart, Monash Health, Clayton, VIC Australia; 2 Department of Cardiology, The Heart Centre, Rigshospitalet, University of Copenhagen, Copenhagen, Denmark; 3 Department of Cardiology, Aarhus University Hospital, Skejby, Aarhus, Denmark

**Keywords:** Imaging, Coronary disease, Ischemia, Computed tomography, Fractional flow reserve, Myocardial perfusion imaging

## Abstract

Computed tomography derived fractional flow reserve (FFR_CT_) and computed tomography stress myocardial perfusion imaging (CTP) are techniques to assess haemodynamic significance of coronary stenosis. To compare the diagnostic performance of FFR_CT_ and static rest/stress CTP in detecting fractional flow reserve (FFR) defined haemodynamically-significant stenosis (FFR ≤ 0.8). Fifty-one patients (96 vessels) with suspected coronary artery disease from a single institution planned for elective invasive-angiography prospectively underwent research indicated 320-detector-CT-coronary-angiography (CTA) and adenosine-stress CTP and invasive FFR. Analyses were performed in separate core-laboratories for FFR_CT_ and CTP blinded to FFR results. Myocardial perfusion was assessed visually and semi-quantitatively by transmural perfusion ratio (TPR). Invasive FFR ≤ 0.8 was present in 33% of vessels and 49% of patients. FFR_CT_, visual CTP and TPR analysis was feasible in 96%, 92% and 92% of patients respectively. Overall per-vessel sensitivity, specificity and diagnostic accuracy for FFR_CT_ were 81%, 85%, 84%, for visual CTP were 50%, 89%, 75% and for TPR were 69%, 48%, 56% respectively. Receiver-operating-characteristics curve analysis demonstrated larger per vessel area-under-curve (AUC) for FFR_CT_ (0.89) compared with visual CTP (0.70; p < 0.001), TPR (0.58; p < 0.001) and CTA (0.70; p = 0.0007); AUC for CTA + FFR_CT_ (0.91) was higher than CTA + visual CTP (0.77, p = 0.008) and CTA + TPR (0.74, p < 0.001). Per-patient AUC for FFR_CT_ (0.90) was higher than visual CTP (0.69; p = 0.0016), TPR (0.56; p < 0.0001) and CTA (0.68; p = 0.001). Based on this selected cohort of patients FFR_CT_ is superior to visually and semi-quantitatively assessed static rest/stress CTP in detecting haemodynamically-significant coronary stenosis as determined on invasive FFR.

**Electronic supplementary material:**

The online version of this article (10.1007/s10554-019-01658-x) contains supplementary material, which is available to authorized users.

## Introduction

Ischemia assessment remains the cornerstone management of stable coronary artery disease (CAD), as its presence and burden predicts benefit from revascularization [1, 2]. While CT coronary angiography (CTA) in its current form cannot reliably predict lesion specific ischemia, novel techniques such as CT stress myocardial perfusion imaging (CTP) and non-invasive CT-derived fractional flow reserve have been demonstrated to accurately predict lesion specific ischemia as determined by invasive fractional flow reserve (FFR) [3, 4]. These techniques differ widely in physiological principles, acquisition requirements, image processing and result interpretation.

Ischemia detection in CTP requires two scans which are performed during rest and vasodilator stress. When significant ischemia is present, the lack of blood flow during vasodilator stress results in lower contrast attenuation in the distal subtended myocardium, which can be determined using static or dynamic acquisition. In static CTP, perfusion defects are typically detected visually or semi-quantitatively using the transmural perfusion ratio (TPR). In dynamic CTP, perfusion can be quantified using mathematical algorithms [5].

FFR_CT_ is defined as the ratio of the CT-derived distal coronary and aortic pressure [6]. Pressure can be derived by computational fluid modeling applied on standard CT angiogram datasets with no need of additional medication, contrast or radiation. The fluid modeling is performed centrally using a supercomputer in a remote location with the test result available within 4–24 h.

The techniques of CTP and FFR_CT_ are used in selected centres in the United States. The aim of this paper is to compare the diagnostic performance of static CTP and FFR_CT_ to detect lesion specific ischemia, using the established outcome-based reference standard of invasive FFR [7].

## Methods

Symptomatic patients with no known CAD who were at intermediate or high risk and were scheduled for clinically mandated invasive coronary angiography were prospectively screened and enrolled from a single institution. Patients were excluded for age < 40 years, advanced atrioventricular block, atrial fibrillation, recent myocardial infarction, severe left main disease, renal insufficiency (estimated glomerular filtration rate < 60 ml min/1.73 m^2^), bronchospastic lung disease requiring long term steroid therapy and contraindications to iodinated contrast or adenosine. All enrolled patients underwent research indicated 320-detector CTA, stress static CTP and invasive FFR, which was undertaken in at least 1 major epicardial vessel with > 2 mm diameter, with a visually assessed stenosis between 10 and 90% during invasive angiography. The study was approved by the institutional human research ethics committee and all participants provided written informed consent.

### CT imaging protocol

Cardiac CT assessment was performed using a 320-row detector CT scanner (Aquilion One Vision; Canon Medical Systems Corp, Otawara, Japan). The scan protocol consists of a calcium score scan, a rest CTA followed by a stress CTP 20 min later [8]. Patients were requested to refrain from caffeine 8 h prior to the scan. Prior to the rest CTA, patients received sublingual nitoglycerin (400 mcg) and beta-blockers were administered to achieve a pre-CTA scan heart rate of 60 beats per minute. Intravenous adenosine (140 mcg/kg/min) was administered for 3 min prior to stress CTP scan acquisition. Scan parameters were: detector collimation 320 × 0.5 mm; tube current 300–500 mA (depending on body mass index); tube voltage 120 kV if BMI ≥ 25 (100 kV if BMI < 25); temporal resolution of 135 ms and 75 mls of contrast were used in both rest CTA and stress CTP. Prospective ECG gated CT scans were performed at rest and during vasodilator stress targeting 70–99% of the R–R interval. In event of heart rates > 70 bpm during stress CTP, a wider window of acquisition between 30 and 99% was performed. CTA and CTP were triggered in the arterial phase at the predicted peak of contrast bolus for myocardial enhancement [9].

### CTA analysis

The rest CTA analysis was performed in a core CTA laboratory (The Heart Centre, Rigshospitalet, Denmark) by two interpreters (JL, MS) who were blinded to which vessels were interrogated with invasive FFR, and the results of FFR and ICA. All coronary segments ≥ 2 mm were visually analysed and scored for degree of luminal stenosis on a dedicated workstation (Vitrea Fx, 6.4, Vital Images, USA) in accordance to the 18 segment coronary model [10]. A vessel was considered significantly stenotic if there was ≥ 1 segment which was non-evaluable or with a ≥ 50% luminal narrowing.

### CTP analysis

Assessment of stress CTP was performed using both rest and stress images in a core laboratory (The Heart Centre, Rigshospitalet, Denmark) by two experienced interpreters (JL 8 years, MS 5 years) who were blinded to which vessels were interrogated with invasive FFR, and the results of FFR and ICA with disagreement resolved by consensus.

The images were reconstructed at 3% phase intervals to facilitate CTP interpretation using FC03 reconstruction kernel [8]. The phase with the least cardiac motion was selected, and images were interpreted using a narrow window width and level setting (W300/L150), and an averaged multiplanar reconstruction slice thickness of 3 to 5 mm.

First each of the 17 myocardial segments, according to the American Heart Association myocardial segment model [11], was matched to a major epicardial vessel, and visually assessed on rest and stress static CTP for the absence or presence of a reversible perfusion defect. A vessel was considered significant if a reversible perfusion defect was present in ≥ 1 of its matched myocardial segments. When artifacts were present in > 50% of the vessel-subtended myocardial segments, the vessel was deemed uninterpretable. Reader confidence was recorded for interpretation. Reader confidence of each vessel territory was scored on a scale of 1 to 5, 1 = very uncertain, 2 = uncertain, 3 = fair, 4 = good, 5 = excellent.

For the quantitative assessment, the transmural perfusion ratio was scored for each myocardial segment using custom analysis software (Vitrea Fx 6.4, Vital Images) using the same phase of images chosen for visual analysis. An automated border detection algorithm was applied to define the subendocardial and subepicardial borders after manual adjustments to the left ventricular axis and myocardial contouring. The myocardium is divided into three myocardial layers—the subendocardium, mid myocardium and subepicardial layers, and the attenuation density in each layer was calculated. Transmural perfusion ratio is calculated as the ratio of the segment specific subendocardial attenuation density to the mean attenuation density of the entire subepicardial layer of any given short axis slice. The segment with the lowest transmural perfusion ratio value was chosen to represent perfusion for each major vessel.

### FFR_CT_ analysis

The CTA dataset was sent for FFR_CT_ analysis at HeartFlow Inc., Redwood City, USA. FFR_CT_ analysis was completed using the most recent generation of FFR_CT_ analysis software. First, a CT-derived luminal model of the coronary tree is constructed, second, physiological assumptions are applied to predict coronary inlet and outlet flow, pressure and microvascular resistance during maximal hyperemia [6]. Finally a numerical solution provided by a parallel supercomputer onto a three dimensional CT-derived mesh model is used to compute coronary pressures hence FFR_CT_ along the coronary tree. A luminal model of FFR_CT_ in the entire coronary tree is generated. The FFR_CT_ value for each vessel is taken as that occurring at the corresponding position of the pressure wire sensor recorded during invasive FFR measurement. A vessel with FFR_CT_ ≤ 0.8 was considered significant.

### Invasive coronary angiography and FFR

Invasive FFR was performed in the distal coronary artery as per standard practice after administration of intracoronary nitroglycerin (100mcg). It was performed in at least 1 vessel segment with diameter ≥ 2 mm with visual stenosis between 10 and 90%. Decisions regarding which vessel was interrogated with invasive FFR was at the discretion of the interventionist, who were blinded to the CT findings. The FFR value was recorded during steady state after administration of intravenous adenosine at 140 mcg/kg/min. Pullback of the pressure sensor back into the tip of the guiding catheter was performed, and only vessels with recording of a signal drift in FFR of < 0.05 were included. An FFR value of ≤ 0.8 was chosen to define hemodynamically significant stenosis.

### Endpoints

The primary endpoint was per vessel diagnostic performance as assessed by the area under the receiver-operating characteristic curve (AUC) of FFR_CT_, visually assessed CTP and TPR using invasive FFR ≤ 0.8 as reference standard to define hemodynamically significant stenosis. Secondary endpoints included (1) per vessel and per patient diagnostic accuracy, sensitivity, specificity, positive predictive value (PPV) and negative predictive value (NPV), (2) combined per vessel CTA + visual CTP, CTA + TPR and CTA + FFR_CT_ as assessed by AUC and (3) per patient diagnostic performance as assessed by AUC. Primary and secondary analyses was performed on all interpretable vessels for each technique, after excluding uninterpretable vessels.

### Statistical analyses

Continuous variables are presented as mean ± SD if normally distributed whereas categorical variables are expressed as percentage. The Wilcoxon signed rank test was used to compare the median FFR values. The correlation between FFR_CT_ and invasive FFR values was determined with a Pearson Correlation coefficient. The AUC comparisons were performed on per vessel and per patient level according to the method described by DeLong et al. [12], treating FFR_CT_ and TPR as continuous variables and visual CTP as dichotomous variable. Patient identity was included as a cluster variable to account for multiple arteries taken from individuals. The optimal TPR threshold (0.94) for detecting FFR defined hemodynamically significant stenosis was determined using the Youden Index [13]. The incremental diagnostic value of adding FFR_CT_, CTP or TPR to CTA was assessed by AUC using a binary logistic regression model. Inter-observer variability was assessed for FFR_CT_, TPR, CTP treated as dichotomized variables using kappa statistics on 15 randomly selected patients including 30 vessel territories. Additional Bland Altman plot analysis was performed for FFR_CT_ and TPR to evaluate inter-observer reproducibility. Statistical analysis was performed using SPSS version 24. A p value < 0.05 was considered statistically significant.

## Results

**Fig. 1:**
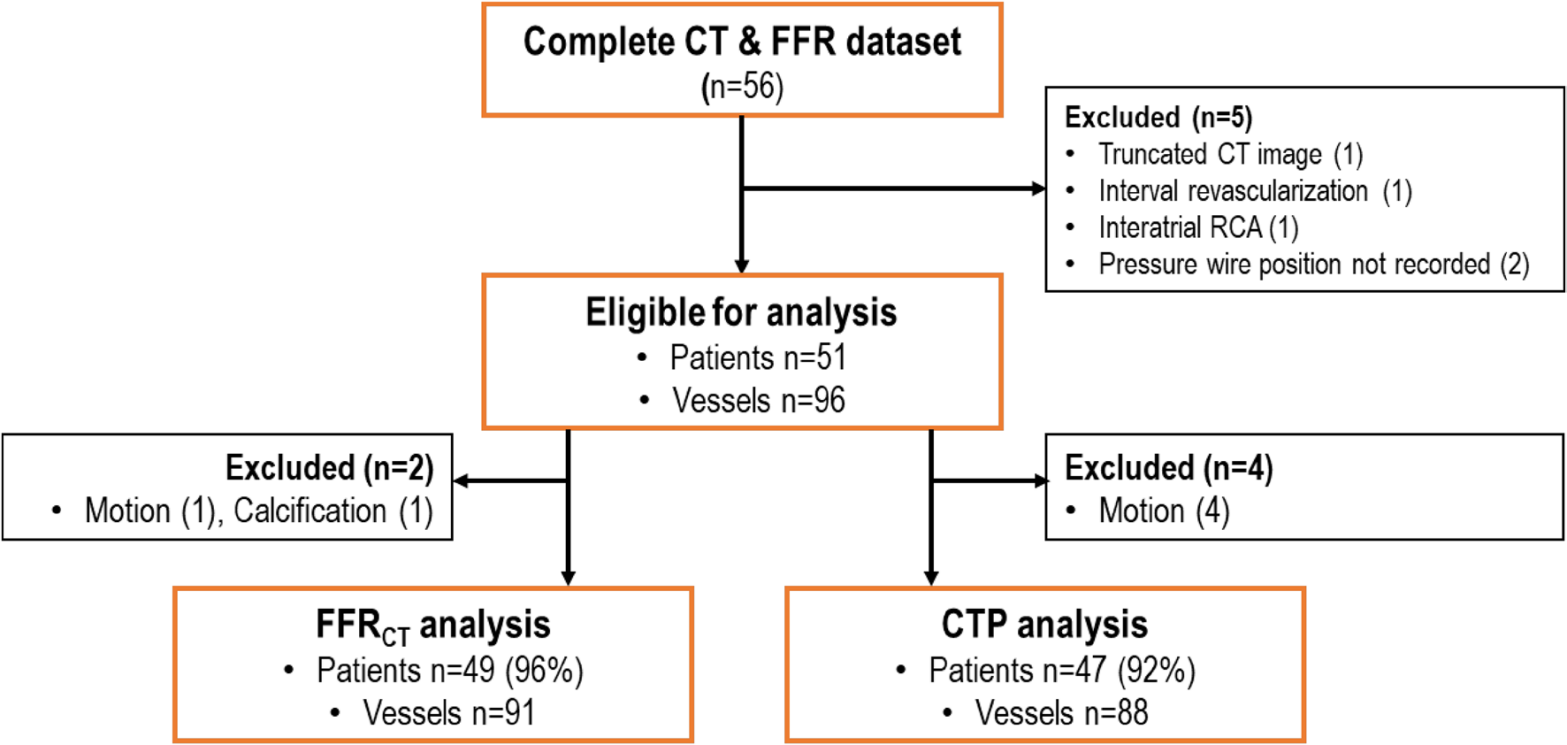
Patient enrollment chart

Patient enrollment flow chart is illustrated in Fig. 1. Among the 56 patients who underwent CTA, CTP and invasive FFR, 5 patients were excluded due to truncated image [1], interatrial RCA (n = 1), interval revascularization (n = 1), and pressure wire position not recorded (n = 2). Of the remaining 51 patients (including 96 FFR-interrogated vessels) deemed eligible for analysis, mean age was 61.9 ± 9.8 years and 76.5% were male. Patient and vessel characteristics and CT scan parameters are summarized in Tables 1, 2 and 3.

**Table 1:** Patient characteristics

	N = 51 patients
Age, mean ± SD (N)	61.9 ± 9.8
Male	76.5% (39/51)
Diabetes mellitus	29.4% (15/51)
Hypertension^a^	76.5% (39/51)
Hyperlipidemia^b^	86.2% (44/51)
Current smoker	19.6% (10/51)
Former smoker	31.4% (16/51)
Angina type^a^	
Typical	19.6% (10/51)
Atypical	56.8% (29/51)
Non cardiac chest pain	21.6% (11/51)
Updated diamond-forrester risk score, %	
Low and Intermediate pre-test risk	80.4% (41/51)
High pre-test risk	19.6% (10/51)
Body mass index, mean ± SD (N)	27.8 ± 4.6
Creatinine (mmol/L), mean ± SD (N)	79.6 ± 15.4

*SD* standard deviation

^a^Blood pressure > 140/90 mmHg or treatment for hypertension

^b^Total cholesterol > 180 mg/dl or treatment for hypercholesterolemia

**Table 2:** Vessel characteristics

Characteristics	
Calcium score (median, IQR)	318 (90–1012)
Vessel	
Left anterior descending artery	44.8% (43/96)
Left circumflex artery	18.8% (18/96)
Right coronary artery	15.6% (15/96)
Diagonal branch	3.1% (3/96)
Obtuse marginal branch	12.5% (12/96)
RPDA or RPLV branch	4.2% (5/96)
Patients with CTCA maximum stenosis > 50%	70.6% (36/51)
Vessels with CTCA maximum stenosis > 50%	51.0% (49/96)
Patients with FFR_CT_ ≤ 0.80	49.0% (24/49)
Vessels with FFR_CT_ ≤ 0.80	37.4% (34/91)
Patients with perfusion defect in ≥ 1 subtended myocardial segment on visual CTP	42.6% (20/47)
Vessels with perfusion defect in ≥ 1 subtended myocardial segment on visual CTP	25.0% (22/88)
Patients with perfusion defect in ≥ 1 subtended myocardial segment on TPR	63.4% (30/47)
Vessels with perfusion defect in ≥ 1 subtended myocardial segment on visual TPR	65.9% (58/88)
Patients with FFR ≤ 0.80	49.0% (25/51)
Vessels with FFR ≤ 0.80	33.0% (32/96)
Patients with FFR ≤ 0.80 in > 1 vessel	11.8% (6/51)

**Table 3:** Scan characteristics

	CTA	CTP
Nitrates	100%	0%
Beta blockers	92.1%	
kV 100/120	45%/55%	
Radiation exposure (mSv)	4.9 ± 2.2	5.7 ± 3.3
Contrast (ml)	75	75
Heart rate on acquisition	53.4 ± 6.2	66.5 ± 9.6

FFR ≤ 0.8 was present 25 patients (49%) including 32 vessels (33%). Among the patients and vessels eligible for analysis, FFR_CT_ was interpretable in 49 patients (96%) including 91 vessels (91%). FFR_CT_ analysis could not be performed in 2 patients secondary to poor CT image quality. In the first case, there was excessive blooming artefact secondary to vessel calcification and in the second case, there was vessel motion artefact. CTP was interpretable in 47 patients (92%) including 88 vessels (92%). The presence of significant motion artefacts precluded analysis in 4 patients. Mean reader confidence score was 4.2 ± 0.9. The number and percentage of vessels interpreted with reader confidence scores of 1, 2, 3, 4, 5 were 0 (0%), 4 (4.5%), 16 (18.2%), 26 (29.5%), 42 (47.7%) respectively.

**Fig. 2:**
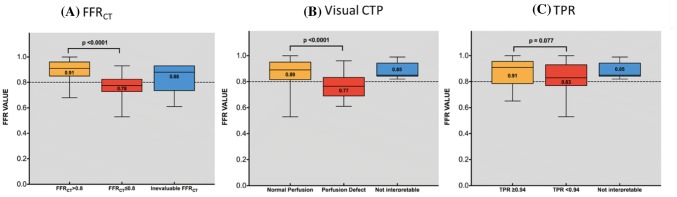
Box plot correlation with FFR. **a** The median FFR in vessels with FFR_CT_ ≤ 0.8 was significantly lower than in vessels with FFR_CT_ > 0.8 (p < 0.0001). **b** Similarly, the median FFR in vessels with visually assessed perfusion defect on CTP was significantly lower than in vessels with normal perfusion (p < 0.0001). **c** The median FFR in vessels with significant TPR was not significantly lower than in vessels with normal TPR (p = 0.08)

**Fig. 3:**
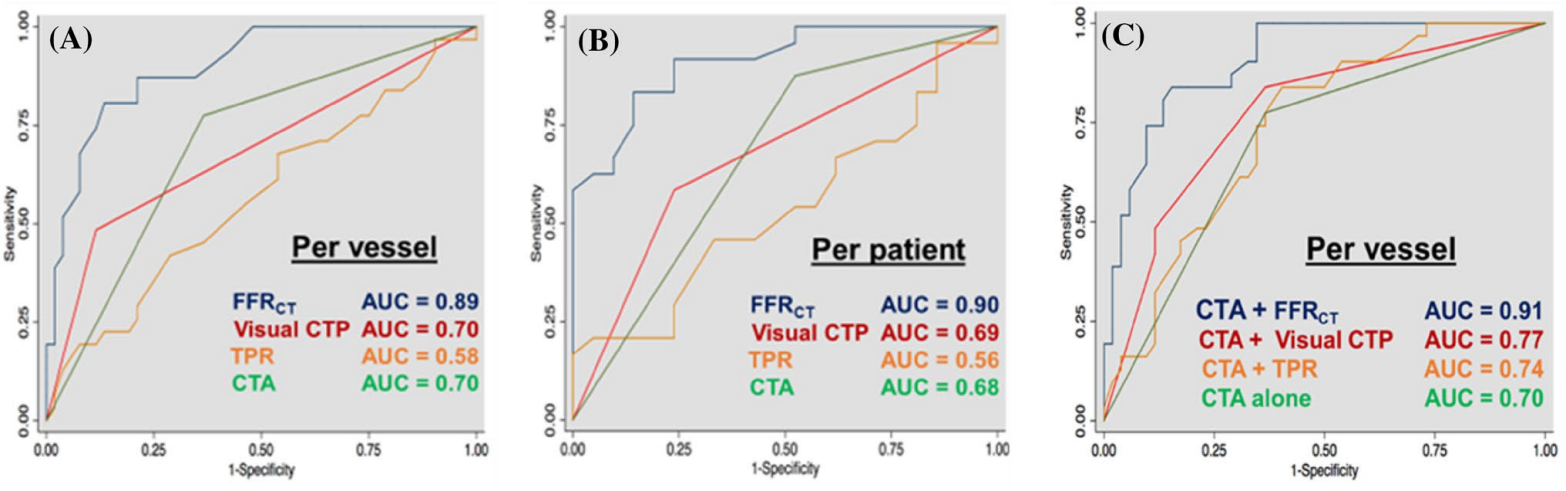
Per vessel and patient diagnostic performance. **a** Per vessel AUC for FFR_CT_ was significantly higher than visual CTP (p < 0.0001), TPR (p < 0.0001) and CTA (p = 0.0007). **b** Per patient AUC for FFR_CT_ was significantly higher than CTP (p = 0.0016), TPR (p < 0.001) and CTA (p = 0.0011). **c** CTA + FFR_CT_ had the highest AUC which was significantly higher than CTA alone (p = 0.0001), CTA + visual CTP (p = 0.0082) and CTA + TPR (p = 0.0009). The AUC for CTA + visual CTP was significantly higher than CTA alone (p = 0.02). The AUC for CTA + TPR was not significantly different from CTA alone (p = 0.26)

**Fig. 4:**
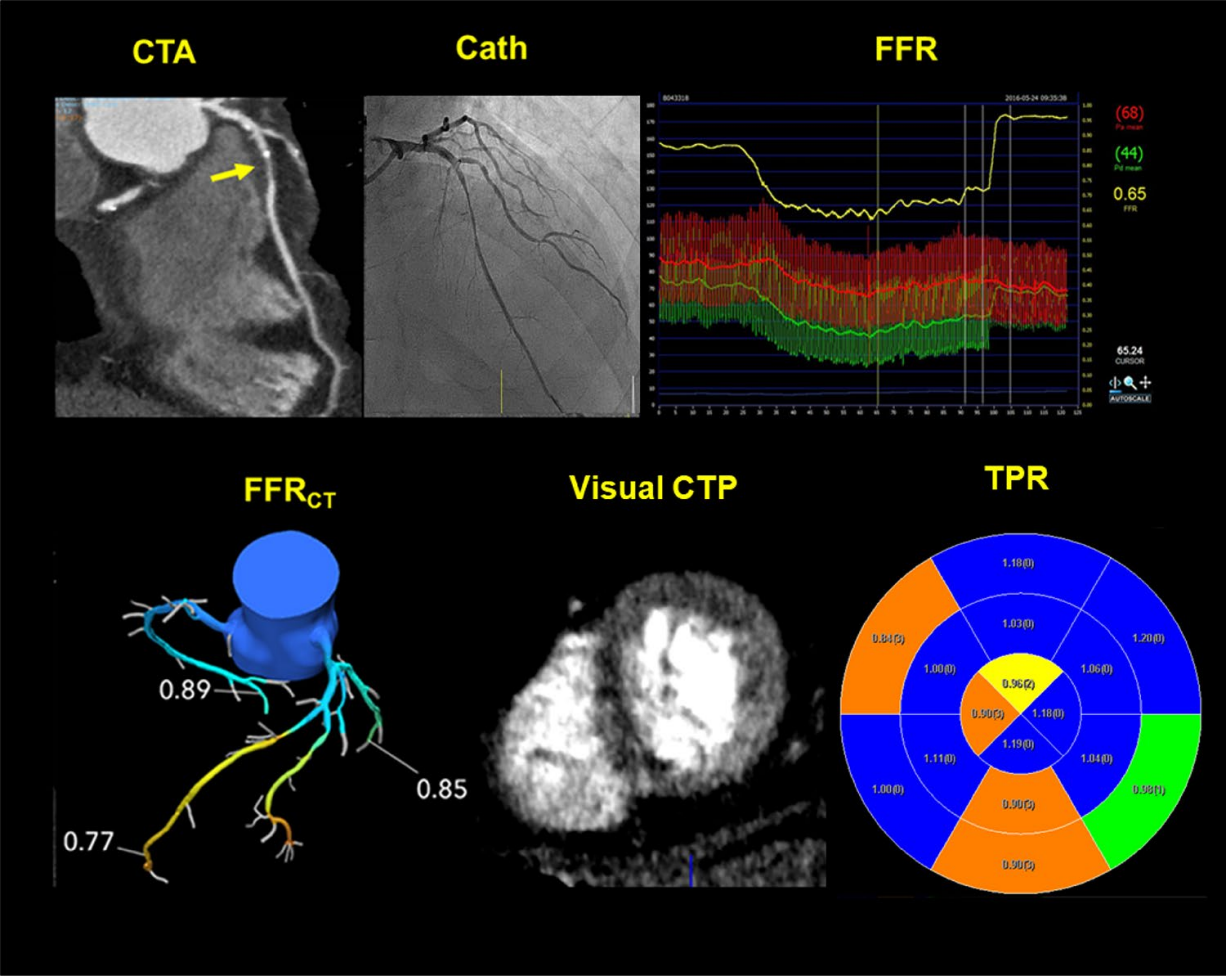
Case example. A 70 year old man with class II angina was identified to have severe mid LAD stenosis on CTA and invasive angiography with invasive FFR of 0.65. FFR was not performed in the remaining arteries which had no significant stenosis. The FFR_CT_ in LAD was 0.77. Perfusion defects were identified visually in the axial images of the myocardium corresponding to the mid anterior and septal segments. The TPR was significant in the basal and distal anterior septum (0.90)

The distribution of FFR_CT_ and FFR is displayed in Supplementary Figure. The box plot of visual CTP, TPR and FFR_CT_ versus invasive FFR is displayed in Fig. 2. The per-patient and vessel diagnostic performance of coronary CTA, CTP, TPR, FFR_CT_ is summarized in Table 4. The per vessel CTA + visual CTP, CTA + TPR and CTA + FFR_CT_ is illustrated in Fig. 3. An example is illustrated in Fig. 4.

**Table 4:** Per vessel and patient diagnostic performance

	CTA > 50	Visual CTP	FFR_CT_	TPR ( < 0.94)
Per vessel analysis (n = 96)				
Included vessels	96	88	91	88
True positive	25	16	25	22
False positive	24	6	9	29
True negative	40	50	51	27
False negative	7	16	6	10
Sensitivity	78.1 (60–90.7)	50.0 (31.9–68.1)	80.6 (62.5–92.5)	68.8 (50.0–83.9)
Specificity	62.5 (49.5–74.3)	89.3 (78.1–96.0)	85.0 (73.4–92.9)	48.2 (34.7–62.0)
PPV	51.0 (36.3–65.6)	72.7 (49.8–89.3)	73.5 (55.6–87.1)	43.1 (29.3–57.8)
NPV	85.1 (71.7–93.8)	75.8 (63.6–85.5)	89.5 (78.5–96.0)	73.0 (55.9–86.2)
Accuracy	67.7	75.0	83.5	55.7
ROC AUC	0.70* (0.61–0.83)	0.70* (0.60–0.79)	0.89 (0.83–0.96)	0.58* (0.45–0.71)
Per patient analysis (n = 51)				
Included patients	51	47	49	47
True positive	22	15	20	17
False positive	14	5	4	13
True negative	12	17	21	8
False negative	3	10	4	9
Sensitivity	88.0 (68.8–97.5)	60.0 (38.7–78.9)	83.3 (62.6–95.3)	68.0 (46.5–85.1)
Specificity	46.2 (26.6–66.6)	77.3 (54.6–92.2)	84.0 (63.0–95.5)	40.9 (20.7–63.6)
PPV	61.1 (43.5–76.9)	75.0 (50.9–91.3)	83.3 (62.6–95.3)	56.7 (37.4–74.5)
NPV	80.0 (51.9–95.7)	63.0 (42.4–80.6)	84.0 (63.9–95.5)	52.9 (27.8–77.0)
Accuracy	66.7	68.1	83.7	53.2
ROC AUC	0.68** (0.56–0.80)	0.69 (0.55–0.82)	0.90 (0.82–0.98)	0.56 (0.40–0.73)

*FFR_CT_ is superior to Visual CTP (p = 0.0001), TPR (p < 0.0001), CTA (p = 0.0007) on per vessel basis

**FFR_CT_ is superior to Visual CTP (p = 0.0016), TPR (p < 0.0001) and CTA (p = 0.0011) on per patient basis

Visual CTP did not reach significance against TPR (p = 0.12) on a per-vessel basis

Visual CTP did not reach significance against TPR (p = 0.22) on a per-patient basis

### Per vessel diagnostic performance

The primary endpoint of per-vessel AUC for FFR_CT_ was 0.89, which was significantly higher than visual CTP (0.70), TPR (0.58) and CTA (0.70) (Fig. 3).

FFR_CT_ had the highest per vessel sensitivity of 80.6% and specificity 85.0%. FFR_CT_ reduced the number of false positive vessels by 63% when compared with CTA. While CTP had high per vessel specificity of 89.3%, the sensitivity was 50.0%. TPR had low sensitivity 68.8% and specificity 48.2%.

The AUC of CTA + FFR_CT_ was 0.91, which was significantly higher than CTA alone (0.70), CTA + visual CTP (0.77) and CTA + TPR (0.74). The AUC for CTA + visual CTP was significantly higher than CTA alone. The AUC for CTA + TPR was not significantly different from CTA alone.

In vessels with CTA > 50%, the AUC for FFR_CT_ was 0.82, which was significantly higher than CTP (0.64, p = 0.025) and TPR (0.51, p = 0.0018). The sensitivity and specificity in this cohort was 79.2% and 72.7% for FFR_CT_. The corresponding numbers for CTP and TPR were 56.0%, 71.4% and 60.0%, 52.4% respectively.

The median per vessel calcium score was 81. In vessels with a calcium score < 81, the diagnostic accuracy of FFR_CT_ was 80.0%. The corresponding values for CTP and TPR was 80.5% and 51.2%. In vessels with a calcium score > 81, the diagnostic accuracy of FFR_CT_ was 89.4%, which was higher than CTP (64.1%) and TPR (48.7%).

### Per patient diagnostic performance

Per patient AUC for FFR_CT_ was 0.90 which was significantly higher than CTP 0.69, TPR 0.56 and CTA 0.68. Per patient accuracy of FFR_CT_ was 83.7% with a sensitivity of 83.3%, specificity 84.0%. Both CTP and TPR had a lower overall accuracy 68.1% and 53.2%, sensitivity 60.0% and 68.0% and lower specificity 77.3% and 40.9% respectively.

Of the 36 patients with at least one significant CTA stenosis, FFR_CT_ was negative in 14 patients (11/14 were truly negative on FFR, 3/14 were falsely negative—mean FFR was 0.75, range 0.68–0.80). The CTP was normal in 15 patients (7/15 were truly negative, 8/15 were falsely negative—mean FFR 0.72, range 0.53–0.80), and TPR was normal in 14 patients (6/14 were truly negative, 8/14 were falsely negative—mean FFR 0.74, range 0.65–0.80).

### Reproducibility

In 15 randomly selected patients, including 30 vessels, per vessel intra-observer variability of FFR_CT_, visual CTP and TPR was modest (k = 0.49, p < 0.01, k = 0.44, p = 0.02 and k = 0.68, p < 0.001 respectively). Bland Altman analysis of FFR_CT_ demonstrated a non-significant mean inter-observer difference of 0.01 (p = 0.12) with standard deviation of 0.039 and 95% limits of agreement of − 0.065 to 0.088. Bland–Altman analysis for TPR demonstrated a mean inter-observer difference of − 0.017 (p = 0.18, 95% limits of agreement: − 0.14 to 0.10).

## Discussion

This study compared the diagnostic performance of static CTP and FFR_CT_ to detect hemodynamically significant stenosis. Both techniques are increasingly used in clinical practice. In this cohort FFR_CT_ is superior to both visual CTP and TPR in diagnostic performance on both per vessel and patient basis. While both visual CTP and FFR_CT_ improved the diagnostic performance of CTA when combined, the improvement was higher when combined with FFR_CT_ compared with CTP. There was no improvement in diagnostic performance of CTA when combined with TPR. Performance of CTP in addition to CTA required use of adenosine, and additional radiation (5.7 mSv on average) and contrast (75 mls).

A recent metanalysis reported the per vessel sensitivity and specificity of static CTP was between 72–82% and 83–88% respectively [14]. However the studies used have not devised a core-laboratory for CTP assessment. Based on this current core-laboratory adjudicated study, the per vessel sensitivity is lower than previously reported at 50%, while the specificity of 89% is within reported range.

A number of studies have compared the diagnostic performance of static and dynamic CTP with point of care CT-FFR using invasive FFR as reference standard [15–17]. Yang et al. demonstrated the AUC of point of care CT-FFR and static CTP acquired using second generation dual source CT were comparably high (0.89 for both) [16]. The sensitivity of static CTP was 79%, specificity was 91%. Coenen et al. in a cohort of 74 patients scanned using second and third generation dual source CT demonstrated comparable AUC of dynamic stress CTP and point of care CT-FFR (AUC = 0.78 for both techniques) [15]. Using an indexed myocardial blood flow threshold, the sensitivity of CTP was 73%, specificity was 68%. Lastly Ihdayhid et al. reported static visual CTP acquired using 320 detector CT had a per vessel sensitivity of 54%. Visual CTP had lower AUC when compared with onsite CT-FFR (0.89 vs. 0.72 p = 0.02) [17]. These results indicate that the diagnostic performance of CTP may potentially vary depending on the scanner used; and whether static or dynamic perfusion assessment is used.

The lower than predicted sensitivity for invasive FFR < 0.8 has been widely reported in stress perfusion techniques [18]. Bettencourt et al. reported per vessel and per patient sensitivity of 55% and 68% respectively in CTP [4]. According to data from meta-analysis and prospective multi-modality comparative trials the per vessel sensitivity of single photon emission computed tomography myocardial perfusion imaging (SPECT MPI) for FFR ≤ 0.8, was found to be between 36 and 57% [18–21] Similarly in the recent Dan-NICAD cohort, magnetic resonance perfusion imaging was found to have a per sensitivity of 41% [21].

This suggests inherent methodological differences in perfusion assessment and FFR_CT_. CTP assesses for the presence of myocardial ischemia which may result from the presence of a significant epicardial stenosis, microvascular dysfunction or both—which may be better accounted by coronary flow reserve (CFR). FFR_CT_, similar to invasive FFR, assesses lesion specific ischemia which results from the presence of a significant epicardial stenosis alone. The use of invasive FFR as the reference standard may hence favour the diagnostic performance of FFR_CT_. Discrepancies between FFR and CFR or myocardial flow as assessed by PET have been described [22, 23]. Accordingly in subjects with an abnormal FFR yet preserved CFR, stress CTP may be normal, despite the presence of a FFR significant stenosis.

There are a number of strengths in this study. First core lab analysis for both CTP and FFR_CT_ was performed blinded to invasive FFR. Notably in similar comparison studies, core lab adjudicated CTP assessment has not been used. Second the study is investigator initiated without support from industry or CT vendor, hence reducing potential bias. Third, static CTP has been assessed using both visual and semi-quantitative methods. Lastly the FFR_CT_ location was matched exactly with the pressure sensor location at time of invasive FFR measurement. The values for FFR_CT_ and invasive FFR changes along the length of coronary artery [24], and meticulous matching assists in discerning true correlation.

There are limitations in this study. (1) This study included limited number of patients enrolled from a single institution. (2) The study cohort included patients referred for clinically mandated elective ICA, and the invasive FFR and CTA/CTP had been performed for research purpose alone. Notably invasive FFR was not performed in all vessels, but at discretion of the interventionist. For this reason, the results may not be generalised to lower risk cohorts with suspected CAD referred for clinically mandated coronary CTA. (3) Our results compared static CTP acquired using 320 detector single source CT with FFR_CT_. These results cannot be conferred to other CTP techniques including dynamic perfusion, dual energy CTP, or CTP performed using other scanner technology including dual source CT. (4) The study only included patients with suspected disease and excluded patients with stenting. Diagnostic performance in these patients, in whom CTP may confer a potential advantage is unknown [25]. (5) Beta blockers and sublingual GTN were administered prior to rest CTA, as had been previously described. These may influence the accuracy of the stress CTP.

## Conclusion

Based on the results of this core lab adjudicated single institution cohort with 320-detector CTA, CTP and invasive FFR, FFR_CT_ demonstrated superior diagnostic performance when compared with visual and semi-quantitatively assessed static CTP for detection of hemodynamically significant stenosis as assessed on invasive FFR on both per vessel and patient basis. While both visual CTP and FFR_CT_ improved the diagnostic performance of CTA when combined, the improvement was higher when combined with FFR_CT_ compared with visual CTP. Semiquantitative TPR perfusion assessment did not improve the diagnostic performance of CTA. Future prospective multicenter core laboratory adjudicated trials may assist in validating these results.

## Electronic supplementary material

Below is the link to the electronic supplementary material.

Supplementary file1 (DOCX 87 kb)

## Abbreviations

CT: Computed tomography
CTA: Computed tomography coronary angiography
FFR_CT_: Non-invasive CT-derived fractional flow reserve
ICA: Invasive coronary angiography
FFR: Fractional flow reserve
CFD: Computational fluid dynamics
ROC: Receiver-operating characteristics curve
AUC: Area under the curve
