# Supplementary material for: Non-invasive CT-derived fractional flow reserve and static rest and stress CT myocardial perfusion imaging for detection of haemodynamically significant coronary stenosis
Source: Int J Cardiovasc Imaging. 2019 Jul 4;35(11):2103–12. doi: 10.1007/s10554-019-01658-x (PMC6805817; doi:10.1007/s10554-019-01658-x)
Supplement: Supplementary file 1 — Supplementary file1 (DOCX 87 kb) [file 10554_2019_1658_MOESM1_ESM.docx]

**Supplementary Figure – FFR_CT_ discrimination for ischemia**

**
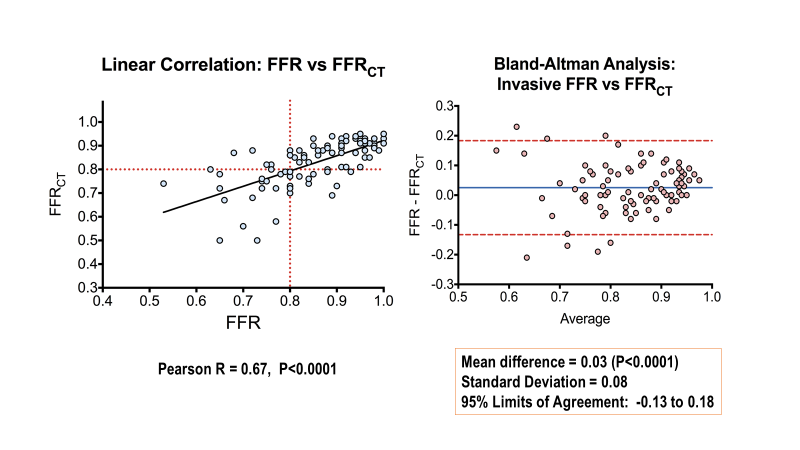
**

There was a moderate linear correlation between FFR_CT_ and invasive FFR. The scatterplot demonstrated greater scatter between FFR_CT_ and invasive FFR at FFR values below 0.80. The Bland Altman plot demonstrated a significant mean difference of 0.03±0.08 (p<0.0001).
